# Infodemiology and hearing: analysis of the search behavior of the Brazilian population

**DOI:** 10.1590/2317-1782/e20240273en

**Published:** 2025-10-17

**Authors:** Ademir Antonio Comerlatto, Mariane Perin da Silva Comerlatto, Iris Layane Santos Santana, Jamille Conceição Santos, Andreza Messias de Arruda, Glória Maria Silva Lima, Kelly da Silva

**Affiliations:** 1 Departamento de Fonoaudiologia, Universidade Federal de Sergipe – UFS - Lagarto (SE), Brasil.; 2 Universidade Federal de Sergipe – UFS - Lagarto (SE), Brasil.

**Keywords:** Infodemiology, Hearing Loss, Tinnitus, Dizziness, Vertigo

## Abstract

**Purpose:**

To investigate the online search behavior of the Brazilian population for information related to hearing loss, tinnitus, and dizziness.

**Methods:**

This ecological and infodemiological study analyzed online search behavior in Brazil for the terms “Hearing Loss,” “Dizziness,” and “Tinnitus” from January 2014 to May 2024, using data from Google Trends™. Normalized relative search volumes (VPR) were calculated, and correlations between the terms were analyzed using Spearman's test, trend analysis with the Mann-Kendall test, and structural breaks with a significance level of 5%.

**Results:**

Significant correlations were found between the terms “Dizziness” and “Hearing Loss,” “Tinnitus” and “Hearing Loss,” and “Tinnitus” and “Dizziness.” An increasing trend in VPR was observed for “Hearing Loss,” “Dizziness,” and “Tinnitus.” Structural breaks for each search term occurred in the following periods: Hearing Loss (two breaks): February 2016 and January 2021; Dizziness (five breaks): July 2015, July 2017, February 2019, April 2020, and December 2021; and Tinnitus (five breaks): April 2015, April 2017, March 2018, April 2020, and April 2022.

**Conclusion:**

The search behavior over the past decade reveals a growing interest in information about hearing loss, tinnitus, and dizziness, with correlated results among the three terms. Sergipe had the highest search volume for “hearing loss,” Maranhão for “dizziness,” and São Paulo for “tinnitus.”

## INTRODUCTION

The Internet has revolutionized the way we access and share various types of content, resulting in an accelerated process of information dissemination. This phenomenon highlights potential trends in popular topics, including those related to health and well-being. In this context, social networks and web browsers have become popular sources for seeking information related to health status, medications, and diagnoses^(1)^.

Within this framework, Infodemiology, the epidemiology of information, emerged as a field within health informatics, aiming to collect information from electronic media as a tool to manage the distribution of health information, assess the quality of these data, and monitor public behavior in response to information. It also seeks to address infodemic phenomena, defined as the abundance of information, accurate or not, that spreads among people through digital and physical information systems^(2)^. Over the past decade, this research methodology has gained traction in health-related fields^(3-7)^.

One of the resources available in infodemiology is Google Trends, a tool provided by Google that enables the analysis of search query volumes within specific regions and timeframes. Analyzing these data can reveal trends and patterns in public interest regarding various health issues^(5)^.

Hearing loss is a topic of significant interest to both the general population and health professionals and policymakers. Hearing loss ranks second among chronic diseases worldwide^(8)^. This condition can affect individuals across all stages of life and impact social interactions, cognition^(9)^, and quality of life^(10)^. Globally, it is estimated that 430 million people, over 5% of the population, have disabling hearing loss. Data published by the World Health Organization (WHO) indicate that this situation may worsen significantly by 2050, with projections suggesting that approximately 2.5 billion people will experience some degree of hearing loss, and at least 700 million will require hearing rehabilitation^(11)^.

In Brazil, the most recent study published by the Brazilian Institute of Geography and Statistics (IBGE) estimates that 1.1% of the population has hearing loss, with 0.9% reporting acquired hearing loss and 0.2% having congenital hearing loss^(12)^.

Other notable auditory health symptoms include tinnitus and dizziness. Tinnitus affects 14% of the global adult population, approximately 740 million people^(13)^. Dizziness, in turn, is common across all age groups, with prevalence ranging from 1.8% in young adults to over 30% in older people^(13)^.

These auditory health issues not only have individual impacts but also represent a significant burden for healthcare systems and global economies. The lack of early diagnosis, appropriate treatment, and rehabilitation can exacerbate these conditions, leading to long-term adverse consequences for both patients and society.

Therefore, this study aims to explore search trends related to hearing loss, tinnitus, and dizziness among the Brazilian population. The results may provide valuable insights for healthcare professionals, researchers, and policymakers. Thus, the objective of this study was to investigate the online search behavior of the Brazilian population for information related to hearing loss, tinnitus, and dizziness.

## METHODS

This ecological and infodemiological time-series study utilized Google Trends™^(14)^ as a proxy to analyze the search behavior and interests of the Brazilian population regarding the terms “Hearing Loss,” “Dizziness,” and “Tinnitus” on Google Search™.

Population interest was measured based on the normalized relative search volume (RSV) over a decade (January 2014 to January 2024). The selected timeframe reflects the significant increase in Internet and mobile phone access among Brazilians in the past decade.

RSV is provided by the Google Trends™ platform, where an RSV of 100 represents the highest popularity of a term within the analyzed period and location. At the same time, other values indicate relative proportions compared to this peak. This normalization accounts for the total number of searches conducted in the selected region and period, ensuring that results reflect proportional interest and are not influenced by differences in overall search volume. This allows for fair and accurate comparisons of trends and seasonality.

To create maps indicating RSV by Brazilian state and the Federal District, the Microsoft OpenStreetMap platform was used. Data analysis involved identifying peaks of interest, generating graphs for result visualization, correlating search terms, and interpreting temporal trends. Data were extracted from the platform in CSV format, tabulated in Microsoft Excel, and statistically analyzed using R software version 4.2.3 (significance level of 5%). RSVs for each search term were collected monthly from January 2014 to May 2024.

To assess the normality of the results, the Shapiro-Wilk test was used, indicating a non-parametric distribution. To examine possible correlations between the RSVs of the studied terms, the Spearman correlation test was performed. For the general trend analysis of each time series, the Mann-Kendall test was conducted.

To detect structural breaks in the studied time series, the change point package in R was used. This package is designed to detect significant changes in a time series. Changes were detected by analyzing shifts in means and variances simultaneously over the decade studied.

For interpreting correlation results, the guidelines of Dancey and Reidy^(15)^ were followed: correlation coefficients up to 0.4 are considered weak, 0.4 to 0.7 moderate, and above 0.7 strong. For interpreting Mann-Kendall test results, values close to +1 indicate a stronger upward trend over the years studied.

## RESULTS

The mean RSV for the term “hearing loss” was 63.7 (±16.1), for “dizziness” it was 62.4 (±20.8), and for “tinnitus” it was 53.1 (±18.4). The lowest RSV for “hearing loss” was 27, occurring in December 2015. The lowest RSVs for “dizziness” and “tinnitus” were 28 (June 2014) and 20 (January 2015), respectively. All terms reached their maximum RSV at some point during the studied time series.

Upon investigating possible correlations among the terms, a significant, positive, and moderate correlation was observed between “dizziness” and “hearing loss,” as well as between “tinnitus” and “hearing loss.” A strong correlation was found between searches for “tinnitus” and “dizziness.” Table 1 presents the results.

**Table 1:** Correlation between search terms

		**Hearing Loss**	**Dizziness**
**Dizziness**	Spearman’s rho	0.668	-
	p-value	< .001*	-
**Tinnitus**	Spearman’s rho	0.659	0.915
	p-value	< .001*	< .001*

* Statistically significant values

**Figure 1:**
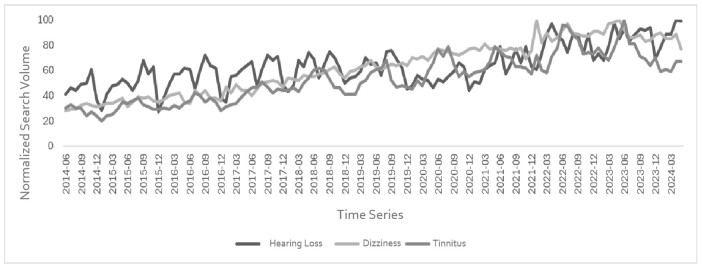
Relative Search Volume for the terms studied over the last decade

**Figure 2:**
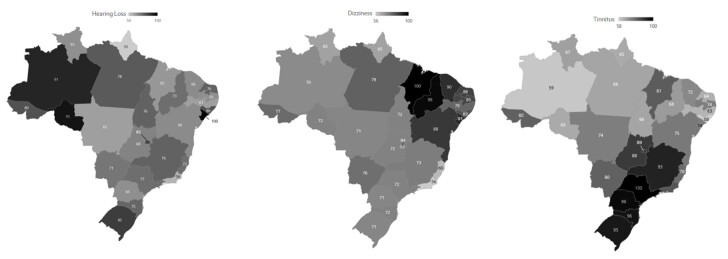
Georeferencing of normalized search volume by Brazilian state and Federal District for the search terms studied

Legend: First Map: Search for Hearing Loss; Second Map: Dizziness; and Third Map: Tinnitus. Darker colors indicate greater Normalized Relative Search Volume

A moderate and significant upward trend was observed for the RSV of “hearing loss” (Tau= 0.52; P<0.01), and a strong upward trend for the RSVs of “dizziness” (Tau= 0.86; p<0.01) and “tinnitus” (Tau= 0.71; p<0.01). Figure 1 displays the RSV values for the target terms throughout the 10-year time series. Figure 2 shows the mean RSV by term over the time series by Brazilian state and the Federal District. The term with the highest search volume was “hearing loss.” Variations in search volume were observed across Brazilian states. The state with the highest search volume for “hearing loss” was Sergipe, for “dizziness” it was Maranhão, and for “tinnitus” it was São Paulo.

**Figure 3:**
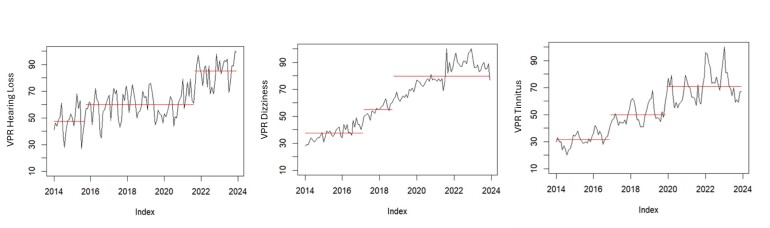
Structural breaks across the time series for the search terms

Two structural breaks were detected in the time series for “hearing loss” (February 2016 and January 2021), five for “dizziness” (April 2015, July 2017, February 2019, April 2020, and December 2021), and five for “tinnitus” (April 2015, April 2017, March 2018, April 2020, and April 2022). Figure 3 presents the graphs with the structural breaks. These breaks represent moments in the time series when significant changes in the statistical structure of the data occurred, indicating shifts in search patterns and potential emerging trends.

Caption: First graph: Hearing Loss searches; Second graph: Dizziness; and Third graph: Tinnitus. RVP: Normalized Relative Search Volume. The red lines indicate periods of change in the population's search behavior

## DISCUSSION

The results of this study underscore the relevance of online search trends as a tool for observing the interests and concerns of the Brazilian population regarding auditory health. The average standardized search values (RSV) for the terms “hearing loss,” “dizziness,” and “tinnitus” demonstrate substantial and sustained interest over time, suggesting a possible interrelationship among these auditory health conditions in public perception. This phenomenon aligns with the growing global concern for auditory health, as indicated by World Health Organization^(11)^ data projecting a significant increase in hearing loss cases by 2050.

The positive and moderate correlations between “dizziness” and “hearing loss,” and between “tinnitus” and “hearing loss,” suggest a frequent association of these symptoms. The strong correlation between “tinnitus” and “dizziness” indicates that these symptoms are often searched together, possibly reflecting a common clinical condition.

Trend analysis reveals a significant increase in interest in the studied terms over the past ten years. A moderate and significant upward trend was observed for the RSV of “hearing loss.” A strong upward trend was also evident for the RSVs of “tinnitus” and “dizziness,” especially from 2020 to 2024, indicating an even more pronounced increase in searches.

The growing trend may be attributed to several factors, including increased internet access and greater awareness of auditory health issues. In 2021, 90.0% of Brazilian households had internet access, representing a six-percentage-point increase compared to 2019, when access was at 84.0%^(12)^. The expansion of internet connectivity in Brazil, coupled with the growth of mobile connectivity, which facilitates real-time internet access and searches, may have facilitated access to information and, consequently, increased the volume of searches related to auditory health.

Another important hypothesis for the increase in searches for these topics over the past four years is the association of the studied terms with the symptoms and sequelae of COVID-19, due to the disease's harmful effects on the auditory system. Some studies and clinical reports suggest that infection with the SARS-CoV-2 virus, which causes COVID-19, may be associated with various auditory problems, including hearing loss, tinnitus, and dizziness^(16-18)^.

Studies indicate that COVID-19 negatively affects the outer hair cells of the cochlea and that auditory system damage can occur due to viral infections, primarily affecting the cochlea but potentially also impacting the auditory brainstem^(19)^. However, one study^(20)^ describe that the prevalence of auditory symptoms, such as sudden or progressive sensorineural hearing loss and tinnitus, is not yet fully clear in patients who have had COVID-19, although their presence may be an early sign of thrombosis or the spread of infection to the brain.

Regarding hearing loss, systematic review studies^(16-20)^, a scoping review^(17)^, and an umbrella review^(21)^ have concluded that different types and degrees of hearing loss can be identified as sequelae of COVID-19 infection, even in asymptomatic patients.

Tinnitus, along with hearing loss, is the tenth most common complaint among patients in the analyzed studies, accounting for 15% of reports^(21)^. A recent study conducted with individuals infected across different age groups showed that, among the top ten symptoms observed, hearing loss or tinnitus was reported by 15% of the sample post-COVID-19 infection^(16)^. Patients with the disease reported experiencing tinnitus, with descriptions ranging from recurrent to sporadic, fluctuating, persistent, pulsatile, and continuous^(22)^.

Regarding searches for the term “dizziness,” studies^(17,20,21)^ have described that balance disorders such as vertigo and dizziness have increased significantly after COVID-19 infection.

Regional analysis of search volumes revealed variations among Brazilian states. Sergipe, Maranhão, and São Paulo had the highest search volumes for “hearing loss,” “dizziness,” and “tinnitus,” respectively. This result may reflect regional differences in auditory health awareness, access to healthcare services, and the prevalence of auditory conditions, warranting further analysis to understand this asymmetry more deeply. These findings highlight regional patterns and reinforce the importance of targeted strategies to address auditory health demands in Brazil.

The detection of structural breaks in the RSVs of the studied terms indicates specific moments when there were abrupt changes in search interest. For “hearing loss,” the breaks in February 2016 and January 2021 may reflect specific events or campaigns that increased public awareness. The multiple breaks for “dizziness” and “tinnitus” between 2015 and 2022 may indicate fluctuations in public interest, possibly influenced by published studies, news about auditory health, or reports of symptoms related to COVID-19.

Online search data can provide valuable insights for healthcare professionals, researchers, and policymakers. The significant correlations and increased search volumes highlight the need to improve education and health resources related to auditory health. Early screening programs, educational campaigns, and increased access to hearing rehabilitation services are effective strategies that can help mitigate the impact of hearing loss and its associated symptoms.

A systematic review^(13)^ discusses that a considerable number of correlation studies have demonstrated the potential of Google Trends data for monitoring health-related phenomena. For example, information about HIV and incidence rates in the United States enabled the construction of a search-based model to predict HIV incidence in subsequent years. Similarly, Google Trends could have been used to indicate the peak of scarlet fever in the United Kingdom five weeks before its occurrence.

Online searches related to hearing loss, tinnitus, and dizziness among the Brazilian population reveal a growing interest over recent years, possibly influenced by the COVID-19 pandemic and increased digital connectivity. The analysis indicates frequent associations among these symptoms and underscores the need for strategies that promote auditory health education, early screening, and access to rehabilitation services.

Given this scenario, a significant responsibility arises for healthcare professionals, the academic community, and especially scientific societies to provide evidence-based content in clear and accessible language for the general public. This approach not only broadens access to quality information but also combats the spread of myths and inaccurate information, strengthening health education and promoting informed choices regarding auditory care.

This study also has limitations. The analysis is based on Google Trends search data, which may not capture all searches performed on other platforms. Additionally, online searches can be influenced by various external factors, such as media coverage and public health campaigns, which may not accurately reflect the prevalence or severity of health conditions.

Future research may benefit from integrating data from other sources, such as social networks and clinical databases, to provide a more comprehensive view of search trends and behaviors. Furthermore, longitudinal studies could explore the underlying reasons for the observed structural breaks and investigate the impact of specific interventions on the auditory health of the population.

## CONCLUSION

Search behavior over the past decade has revealed a growing interest in information on hearing loss, tinnitus, and dizziness, with correlated results among these three terms. Sergipe had the highest search volume for “hearing loss,” Maranhão for “dizziness,” and São Paulo for “tinnitus.”
