# Supplementary material for: Infodemiology and hearing: analysis of the search behavior of the Brazilian population
Source: Codas. 2025 Oct 17;37(5):e20240273. doi: 10.1590/2317-1782/e20240273en (PMC12533532; doi:10.1590/2317-1782/e20240273en)
Supplement: Translation

# Translation: Infodemiologia e audição: análise do comportamento de buscas da população brasileira

**DOI:** 10.1590/2317-1782/e20240273pt

**Authors:** Ademir Antonio Comerlatto, Mariane Perin da Silva Comerlatto, Iris Layane Santos Santana, Jamille Conceição Santos, Andreza Messias de Arruda, Glória Maria Silva Lima, Kelly da Silva

**Affiliations:** 1 Departamento de Fonoaudiologia, Universidade Federal de Sergipe – UFS - Lagarto (SE), Brasil.; 2 Universidade Federal de Sergipe – UFS - Lagarto (SE), Brasil.

**Keywords:** Infodemiologia, Perda Auditiva, Zumbido, Tontura, Vertigem

## Abstract

**Objetivo:**

Investigar o comportamento de busca online por informações relacionadas à perda auditiva, zumbido e tontura pela população brasileira.

**Método:**

Estudo ecológico e infodemiológico analisou o comportamento de busca online no Brasil para os termos “Perda Auditiva”, “Tontura” e “Zumbido” entre janeiro de 2014 e maio de 2024, utilizando dados do Google Trends™. Foram calculados os volumes de pesquisa relativo normalizado (VPR) e analisadas as correlações entre os termos usando o teste de Spearman, a tendência com o teste de Mann-Kendall, e quebras estruturais com nível de significância de 5%.

**Resultados:**

Encontradas correlações significativas entre os termos “Tontura” e “Perda Auditiva”, “Zumbido” e “Perda Auditiva” e “Zumbido” e “Tontura”. Observou-se tendência de crescimento no VPR para “Perda Auditiva”, “Tontura” e “Zumbido”. As quebras estruturais detectadas para cada termo de busca ocorreram nos seguintes períodos: Perda auditiva (duas quebras): fevereiro de 2016 e janeiro de 2021; Tontura (cinco quebras): julho de 2015 e de 2017; fevereiro de 2019; abril de 2020 e dezembro de 2021 e Zumbido (cinco quebras): abril de 2015 e de 2017, março de 2018, abril de 2020 e de 2022.

**Conclusão:**

O comportamento de busca na última década revela um crescente interesse por informações sobre perda auditiva, zumbido e tontura, com resultados correlacionados entre os três termos. Sergipe apresentou o maior volume de buscas por “perda auditiva”, Maranhão por “tontura” e São Paulo por “zumbido”.

## INTRODUÇÃO

A internet revolucionou como acessamos e compartilhamos diversos conteúdos, causando um fenômeno de aceleração no processo de disseminação da informação. Este fenômeno evidencia possíveis tendências de assuntos em alta, inclusive a respeito de temas relacionados à saúde e bem-estar. Aliadas a esse processo, as redes sociais e navegadores da *web* são fontes populares de busca de informações relacionadas ao estado de saúde, medicamentos e diagnósticos^(1)^.

Nesse contexto, surgiu a Infodemiologia, a epidemiologia da informação, como um campo da informática em saúde, visando coletar informação em meios eletrônicos como uma ferramenta para gerir a distribuição de informações sobre saúde, identificar a qualidade desses dados e monitorar o comportamento das pessoas diante das informações. Ainda, visa discutir a infodemia, ou seja, a abundância de informações, precisas ou não, que se espalham entre as pessoas por meio de sistemas de informação digital e física^(2)^. Desde a última década, esta metodologia de pesquisa tem se difundido nas áreas de saúde^(3-7)^.

Um dos recursos à disposição na infodemiologia é o *Google Trends*, uma ferramenta oferecida pelo *Google* que permite analisar o volume de consultas de busca em uma determinada região e período. A análise desses dados pode revelar tendências e padrões de interesse público em relação a diversas questões de saúde^(5)^.

Um assunto de grande interesse populacional e de profissionais e gestores da saúde é a perda auditiva. A perda auditiva ocupa a segunda colocação no ranking das doenças crônicas em âmbito mundial^(8)^. Esta condição pode acometer todos os ciclos de vida e impacta nas interações sociais, na cognição^(9)^ e na qualidade de vida^(10)^. Mundialmente, estima-se que 430 milhões, ou seja, mais de 5% da população é acometida pela perda auditiva incapacitante. Dados publicados pela Organização Mundial da Saúde (OMS), indicam que essa situação pode piorar significativamente até 2050, uma vez que as previsões apontam que cerca de 2,5 bilhões de pessoas terão algum grau de perda auditiva, e pelo menos 700 milhões precisarão de reabilitação auditiva^(11)^.

No Brasil, no último estudo publicado pelo Instituto Brasileiro de Geografia e Estatística (IBGE), estima-se que 1,1% da população possui perda auditiva, sendo que 0,9% referem ter adquirido a perda auditiva e 0,2% possuírem desde o nascimento^(12)^.

No que tange a saúde auditiva, outros sinais e sintomas também se destacam, como a presença de zumbidos e de tontura. O zumbido afeta 14% da população mundial adulta – cerca de 740 milhões de pessoas^(13)^. A tontura, por sua vez, é comum em todas as faixas etárias, com prevalência variando de 1,8% em adultos jovens a mais de 30% em idosos^(13)^.

Esses problemas de saúde auditiva não apenas impactam individualmente, mas também representam um ônus significativo para os sistemas de saúde e economias globais. A falta de diagnóstico precoce, tratamento adequado e reabilitação pode agravar essas condições, levando a consequências adversas a longo prazo para os pacientes e para a sociedade.

Desta forma, este trabalho se propõe a explorar as tendências de buscas relacionadas à perda auditiva, zumbido e tontura pela população brasileira. Estes resultados podem fornecer *insights* valiosos para profissionais de saúde, pesquisadores e formuladores de políticas públicas. Assim, o objetivo deste estudo foi investigar o comportamento de busca online por informações relacionadas à perda auditiva, zumbido e tontura pela população brasileira.

## MÉTODO

Estudo ecológico e infodemiológico, de série temporal que utilizou o *Google Trends*™^(14)^ como proxy para analisar o comportamento de busca e interesse da população brasileira acerca dos termos “Perda Auditiva”, “Tontura” e ”Zumbido” na ferramenta de busca *Google Search*™.

O interesse da população foi mensurado com base no volume de pesquisa relativo normalizado (VPR) em uma década (janeiro de 2014 a janeiro de 2024). A escolha temporal se deve pelo aumento expressivo de acesso à internet e celulares na última década pelos brasileiros.

O VPR é fornecido pela plataforma *Google Trends*™, onde o VPR de 100 representa o momento ou local de maior popularidade do termo no período analisado, enquanto os outros valores indicam proporções relativas em relação a esse pico. Essa normalização considera o total de buscas realizadas na região e no período selecionado, garantindo que os resultados reflitam o interesse proporcional e não sejam influenciados por diferenças no volume total de pesquisas. Isso permite comparações justas e precisas sobre tendências e sazonalidades.

Para a confecção de mapas com a indicação do VPR por estado brasileiro e Distrito Federal foi utilizada a plataforma *OpenStreetMap* da *Microsoft*. A análise dos dados envolveu a identificação de picos de interesse, a criação de gráficos para visualização dos resultados, a correlação entre os termos de busca e a interpretação de tendências temporais. Os dados foram extraídos da plataforma na extensão .CSV e tabulados no software *Microsoft Excel* e analisados estatisticamente no *Software* R versão 4.2.3 (nível de significância de 5%). Foi coletado os VPR para cada termo pesquisado, por mês e ano, no período entre janeiro de 2014 a maio de 2024.

Para análise da normalidade dos resultados, foi utilizado o teste de *Shapiro-Wilk* que indicou se tratar de uma distribuição não-paramétrica. Para verificar as possíveis correlações entre os VPRs dos termos estudados, foi realizado o teste de correlação de *Spearman* e para a análise de tendência geral de cada Série Temporal, foi conduzido o teste de *Mann-kendall*.

Como ferramenta para detectar quebras estruturais nas séries temporais estudadas, foi utilizado o pacote change point do *software* R. Este pacote é projetado para detectar importantes mudanças em uma série temporal. As alterações foram detectadas pela análise de alteração nas médias e nas variâncias, simultaneamente, ao longo da década estudada.

Para interpretação dos resultados de correlação foi considerada a sugestão de Dancey e Reidy^(15)^ em que coeficientes de correlação até 0,4 são considerados fracos, de 0,4 a 0,7 moderados e acima de 0,7 altos. Para interpretação dos resultados do teste de *Mann-kendall* foi considerado que valores próximos de +1 indicam maior força da tendência de crescimento ao longo dos anos estudados.

## RESULTADOS

A média do VPR para o termo perda auditiva foi de 63,7 (±16,1), para o termo Tontura foi de 62,4 (±20,8) e para Zumbido foi 53,1 (±18,4). O menor VPR para Perda auditiva foi de 27 e ocorreu em dezembro de 2015. Já o menor VPR para os termos Tontura e zumbido foram de 28 (junho de 2014) e 20 (janeiro de 2015), respectivamente. Todos os termos obtiveram o VPR máximo obtido em algum momento da série temporal estudada.

Ao investigar possíveis correlações entres os termos, foi observada correlação significativa, positiva e de grau moderado entre Tontura e Perda Auditiva e entre Zumbido e Perda Auditiva. Houve forte correlação entre as buscas por Zumbido e Tontura. A Tabela 1 indica os resultados.

**Tabela 1:** Correlação entre os termos pesquisados

				**Perda Auditiva**		**Tontura**	
**Tontura**		Rho de Spearman		0,668		—	
		p-value		< ,001*		—	
**Zumbido**		Rho de Spearman		0,659		0,915	
		p-value		< ,001*		< ,001*	

* Valores estatisticamente significativos

Foi evidenciada, para os termos de busca, tendência moderada e significativa de crescimento para o VPR do termo Perda Auditiva (Tau= 0,52; P<0,01) e forte tendência de crescimento do VPR para os termos Tontura (Tau= 0,86; p<0,01) e Zumbido (0,71; p<0,01). A Figura 1 apresenta os valores de VPR para os termos alvos deste estudo durante a série temporal de 10 anos. A Figura 2 apresenta o VPR médio, por termo estudado ao longo da série temporal por Estado Brasileiro e pelo Distrito Federal. O termo com maior busca foi o Perda Auditiva. Observou-se variações no volume de buscas de acordo com os estados brasileiros. O estado com maior busca pelo termo Perda auditiva foi o de Sergipe, para tontura foi o do Maranhão e de zumbido foi o estado de São Paulo.

**Figura 1:** Volume de Pesquisa Relativa para os termos estudados ao longo da última década.

**Figura 2:** Georreferenciamento do volume de pesquisa normalizada por estado brasileiro e pelo Distrito Federal para os termos de busca estudados

Legenda: Primeiro Mapa: busca por Perda Auditiva; Segundo mapa: Tontura e Terceiro mapa: Zumbido. Cores mais escuras indicam maior Volume de Pesquisa Relativa Normalizada

Foram detectadas duas quebras estruturais na série temporal relacionada à busca pelo termo Perda Auditiva (fevereiro de 2016 e janeiro de 2021), cinco para o termo Tontura (abril de 2015; julho de 2017; fevereiro de 2019; abril de 2020 e dezembro de 2021) e cinco para o Zumbido (abril de 2015, abril de 2017, março de 2018, abril de 2020 e abril de 2022). A Figura 3 apresenta os gráficos com as quebras estruturais. As quebras estruturais representam os momentos da série temporal em que há mudanças significativas nas estruturas estatísticas dos dados trabalhados, identificando alterações no padrão de busca e possíveis tendências.

**Figura 3:** Quebras estruturais ao longo da série temporal para os termos pesquisados

Legenda: Primeiro gráfico: busca por Perda Auditiva; Segundo gráfico: Tontura e Terceiro gráfico: Zumbido. VPR: Volume de Pesquisa Relativa Normalizada. Os traços vermelhos indicam os períodos de alteração no comportamento de busca da população

## DISCUSSÃO

Os resultados deste estudo destacam a relevância das tendências de busca online como uma ferramenta que permite observar os interesses e preocupações da população em relação à saúde auditiva no Brasil. A média dos valores padronizados de pesquisa (VPR) para os termos “perda auditiva”, “tontura” e “zumbido” demonstra um interesse substancial e contínuo ao longo do tempo, sugerindo uma possível inter-relação entre essas condições de saúde auditiva na percepção pública. Este fenômeno é consistente com a crescente preocupação global com a saúde auditiva, como indicado por dados da Organização Mundial da Saúde^(11)^ que preveem um aumento significativo nos casos de perda auditiva até 2050.

A correlação positiva e moderada entre “tontura” e “perda auditiva” e entre “zumbido” e “perda auditiva” sugere uma associação frequente desses sintomas. A forte correlação entre “zumbido” e “tontura” indica que esses sintomas são frequentemente buscados em conjunto, possivelmente refletindo uma condição clínica comum.

A análise das tendências de crescimento revela um aumento significativo no interesse pelos termos estudados ao longo dos últimos dez anos. Evidenciou-se uma tendência moderada e significativa de crescimento para o VPR do termo Perda Auditiva . Também é possível observar forte tendência de crescimento do VPR para os termos Zumbido e Tontura, especialmente entre os anos de 2020 a 2024, indicando uma busca ainda mais significativa.

A tendência crescente pode ser atribuída a diversos fatores, incluindo o aumento do acesso à internet e a maior conscientização sobre problemas de saúde auditiva. Em 2021, 90,0% dos domicílios no Brasil estavam conectados à Internet, representando um crescimento de 6 pontos percentuais em comparação a 2019, quando o acesso era de 84,0%^(12)^. A expansão da conectividade à internet no Brasil, aliada ao crescimento da conectividade móvel, que facilita o acesso à internet e a realização de buscas em tempo real, pode ter facilitado o acesso à informação e, consequentemente, resultou no aumento do volume de buscas relacionadas à saúde auditiva.

Outra importante hipótese para o aumento do número de buscas desses assuntos nos últimos 4 anos é a associação dos termos estudados com os sintomas e sequelas da COVID-19 devido aos efeitos nocivos da doença no sistema auditivo. Alguns estudos e relatos clínicos sugerem que a infecção pelo vírus SARS-CoV-2, que causa a COVID-19, pode estar associada a diferentes problemas auditivos, incluindo perda de audição, zumbido e tontura^(16-18)^.

Estudos indicam que a COVID-19 afeta negativamente as células ciliadas externas da cóclea e que danos no sistema auditivo podem ocorrer devido a infecções virais, afetando principalmente a cóclea, mas também podendo impactar o tronco cerebral auditivo^(19)^. No entanto, o estudo^(20)^ descreve que a prevalência de sintomas auditivos, como perda auditiva neurossensorial súbita ou progressiva e zumbido, ainda não está totalmente clara em pacientes que apresentaram COVID-19, embora sua presença possa ser um sinal precoce de trombose ou disseminação da infecção para o cérebro.

Quanto à perda auditiva, estudos de revisão sistemática^(16,20)^, uma revisão de escopo^(17)^ e uma revisão guarda-chuva (*umbrella review*)^(21)^ concluíram que diferentes tipos e graus da perda auditiva podem ser identificados como sequelas da infecção por COVID-19 mesmo em pacientes assintomáticos.

O zumbido, juntamente com a perda auditiva, é a 10ª maior queixa entre os pacientes dos estudos analisados, representando um percentual de 15% dos relatos^(21)^. Um estudo recente conduzido com pessoas infectadas em diferentes faixas etárias mostrou que, dentre os 10 principais sintomas observados, a perda auditiva ou o zumbido foram relatados por 15% da amostra pós-infecção por COVID-19^(16)^. Pacientes com a doença relataram experienciar zumbido, com descrições variando de zumbido recorrente, esporádico, flutuante, persistente, pulsátil e contínuo^(22)^

No que se refere aos dados da busca com o termo tontura, estudos^(17,20,21)^ descrevem que alterações do equilíbrio corporal como vertigem e tontura tiveram aumento significativo após a infecção por COVID-19.

A análise regional das buscas revelou variações entre os volumes de buscas dos estados brasileiros. Sergipe, Maranhão e São Paulo foram os estados com maior volume de buscas para “perda auditiva”, “tontura” e “zumbido”, respectivamente. Este resultado pode refletir diferenças regionais na conscientização sobre saúde auditiva, acesso a serviços de saúde e prevalência de condições auditivas, carecendo de maiores análises para entender profundamente esta assimetria. Esses achados destacam padrões regionais e reforçam a importância de estratégias específicas para atender às demandas de saúde auditiva no Brasil.

A detecção de quebras estruturais nos VPRs dos termos pesquisados indica momentos específicos em que houve mudanças abruptas no interesse de busca. Para “perda auditiva”, as quebras em fevereiro de 2016 e janeiro de 2021 podem refletir eventos ou campanhas específicas que aumentaram a conscientização pública. As múltiplas quebras para “tontura” e “zumbido” entre 2015 e 2022 podem indicar flutuações no interesse público, possivelmente, este aumento foi influenciado por publicações de estudos, notícias sobre saúde auditiva ou relatos de sintomas relacionados à COVID-19.

Os dados de busca online podem fornecer insights valiosos para profissionais de saúde, pesquisadores e formuladores de políticas. A correlação significativa e o aumento nas buscas destacam a necessidade de melhorar a educação e os recursos de saúde relacionados à saúde auditiva. Programas de rastreamento precoce, campanhas educativas e aumento do acesso a serviços de reabilitação auditiva são estratégias que podem ser implementadas para mitigar o impacto da perda auditiva e seus sintomas associados.

Uma revisão sistemática^(13)^, discute que um número considerável de estudos de correlação demonstrou o potencial dos dados do Google Trends para serem usados na vigilância de fenômenos relacionados à saúde. Informações sobre HIV e taxas de incidência nos EUA, permitiram construir um modelo baseado em pesquisas para prever a incidência de HIV em anos posteriores. Bem como, o Google Trends poderia ter sido usado para prever o pico da escarlatina no Reino Unido cinco semanas antes de sua chegada.

As buscas online relacionadas à perda auditiva, zumbido e tontura pela população brasileira revelam um crescente interesse ao longo dos últimos anos, possivelmente influenciado pela pandemia de COVID-19 e pelo aumento da conectividade digital. A análise indica associações frequentes entre esses sintomas e destaca a necessidade de estratégias que promovam educação em saúde auditiva, rastreamento precoce e acesso a serviços de reabilitação.

Diante desse cenário, surge uma importante responsabilidade para os profissionais da saúde, a comunidade acadêmica e, especialmente, as sociedades científicas disponibilizarem conteúdo baseados em evidências científicas em uma linguagem clara e compreensível para o público geral. Essa abordagem não apenas amplia o acesso à informação de qualidade, mas também combate a disseminação de mitos e informações imprecisas, fortalecendo a educação em saúde e promovendo escolhas informadas sobre o cuidado auditivo.

Este estudo também possui limitações, a análise baseia-se em dados de busca do *Google Trends*, que pode não capturar todas as buscas realizadas em outras plataformas. Além disso, as buscas online podem ser influenciadas por vários fatores externos, como a cobertura da mídia e campanhas de saúde pública, que podem não refletir com precisão a prevalência ou a gravidade das condições de saúde.

Futuras pesquisas podem se beneficiar da integração de dados de outras fontes, como redes sociais e bancos de dados clínicos, para fornecer uma visão mais abrangente das tendências e comportamentos de busca. Além disso, estudos longitudinais poderiam explorar as razões subjacentes às quebras estruturais observadas e investigar o impacto de intervenções específicas na saúde auditiva da população.

## CONCLUSÃO

O comportamento de busca na última década revela um crescente interesse por informações sobre perda auditiva, zumbido e tontura, com resultados correlacionados entre os três termos. Sergipe apresentou o maior volume de buscas por “perda auditiva”, Maranhão por “tontura” e São Paulo por “zumbido”.
